# Evaluation of early hospital discharge after allogeneic bone marrow transplantation for chronic myeloid leukemia

**DOI:** 10.1590/S1516-31802007000300009

**Published:** 2007-05-03

**Authors:** José Eduardo Nicolau, Leila Maria Magalhães Pessoa de Melo, Daniel Sturaro, Rosaura Saboya, Frederico Luiz Dulley

**Keywords:** Chronic myeloid leukemia, Bone marrow transplantation, Retrospective studies, Ambulatory care, Length of stay, Leucemia mielóide crônica, Transplante de medula óssea, Estudos retrospectivos, Assistência ambulatorial, Tempo de internação

## Abstract

**CONTEXT AND OBJECTIVE:**

The increasing number of patients waiting for bone marrow transplantation in our service led to the implement of an early hospital discharge program with the intention of reducing the interval between diagnosis and transplantation. In this study we analyzed the results from early discharge, with outpatient care for patients with chronic myeloid leukemia who underwent allogeneic bone marrow transplantation.

**DESIGN AND SETTING:**

Retrospective study at the Bone Marrow Transplantation Unit of Hospital das Clínicas, Faculdade de Medicina da Universidade de São Paulo.

**METHODS:**

We compared clinical outcomes within 100 days post-transplantation, for 51 patients with chronic myeloid leukemia (CML) who received partially outpatient-based allogeneic hematopoietic stem cell transplantation, and the results were compared with a historical control group of 49 patients who received inpatient-based hematopoietic stem cell transplantation.

**RESULTS:**

There were significantly fewer days of hospitalization (p = 0.004), *Pseudomonas*-positive cultures (p = 0.006) and nausea and vomiting of grade 2-3 (p < 0.001) in the outpatient group. There were no significant differences in mortality between the groups and no deaths occurred within the first 48 days post-transplantation in the outpatient group.

**CONCLUSIONS:**

This partially outpatient-based hematopoietic stem cell transplantation program allowed an increased number of transplantations in our institution, in cases of CML and other diseases, since it reduced the median length of hospital stay without increasing morbidity and mortality.

## RESUMO

**CONTEXTO E OBJETIVO:**

O número crescente de pacientes com indicação de transplante de medula óssea levou à implantação da alta hospitalar precoce em nosso serviço, com o intuito de reduzir o intervalo entre o diagnóstico e o transplante. Neste trabalho, avaliamos os resultados da alta precoce, com acompanhamento ambulatorial dos pacientes submetidos ao transplante de medula óssea alogênico portadores de leucemia mielóide crônica.

**TIPO E ESTUDO E LOCAL:**

Estudo retrospectivo, realizado no Serviço de Transplante de Medula Óssea do Hospital das Clínicas da Faculdade de Medicina da Universidade de São Paulo.

**MÉTODOS:**

Foram avaliados os resultados do transplante de medula óssea alogênico, de doadores aparentados, até o dia 100 pós-transplante, de 51 pacientes portadores de leucemia mielóide crônica que receberam alta precoce, antes da pega medular. Os resultados foram comparados com o controle histórico constituído por 49 pacientes que receberam alta somente após a pega medular.

**RESULTADOS:**

Houve significativamente menos dias de hospitalização (p = 0,004), culturas positivas para *Pseudomonas sp*. (p = 0,006) e náusea e vômitos graus 2-3 (p < 0,001) no grupo de alta precoce. Não houve diferença significativa entre os grupos quanto à mortalidade e não ocorreu nenhum óbito até o dia 48 pós-transplante no grupo de alta precoce.

**CONCLUSÕES:**

O programa de alta precoce permitiu aumento do número de transplantes em leucemia mielóide crônica e outras doenças em nosso serviço, com redução do número de dias de internação hospitalar sem aumento da morbidade ou da mortalidade.

## INTRODUCTION

Allogeneic hematopoietic stem cell transplantation is considered to be a curative approach towards several diseases such as chronic myeloid leukemia (CML), since it can lead to molecular remission of the BCR-ABL gene.^1–3^ Nevertheless, the high costs relating to this procedure and a lack of hospital beds may impair its utilization in many institutions. A new strategy to overcome these obstacles is to perform hematopoietic stem cell transplantation totally or partially within the outpatient setting, because there is a correlation between length of hospital stay and costs.^4–7^ Some institutions have successfully demonstrated reduced costs when performing autologous hematopoietic stem cell transplantation on an outpatient basis, without an increase in infections, toxicity or mortality rates.^8–13^

In the light of the results obtained with autologous transplantation, some groups have adopted the same procedure for allogeneic transplantation and have concluded that out-patient-based allogeneic hematopoietic stem cell transplantation can be safely performed without protective isolation, yet with a satisfactory outcome.^11,14,15^ Following allogeneic hematopoietic stem cell transplantation, patients discharged to continue their treatment at home during the neutropenic phase have presented less bacteremia, fewer days on intravenous antibiotics, lower transplant-related mortality and lower overall costs, in comparison with patients who remained in hospital.^16,17^

With this new approach, more patients can benefit from this therapeutic procedure, since costs are reduced and more hospital beds are made available. This is of particular interest for developing countries, where there is lack of resources for public healthcare. On the basis of these data, and considering that many patients referred to our center had to wait a long time for transplantation, we decided to implement a partially outpatient-based hematopoietic stem cell transplantation program, with daily outpatient following discharge from hospital, before engraftment, in order to reduce the interval between diagnosis and transplantation.

## OBJECTIVE

The aims of this study were to analyze the results from early hospital discharge following bone marrow transplantation, with supportive outpatient care for patients with CML and to compare the results with a historical control.

## PATIENTS AND METHODS

From October 2000 to October 2002, 51 patients with CML (Philadelphia chromosome-positive) who received a sibling donor allogeneic bone marrow transplant at Hospital das Clínicas, Faculdade de Medicina da Universidade de São Paulo (FMUSP), Brazil, were analyzed. Of these, 45 patients were in the first chronic phase, four in the accelerated phase and two in the blastic phase. Patients were excluded from the analysis in cases of second transplants, peripheral blood stem cell transplants, non-related donor transplants and previous imatinib mesylate treatment.

Patient and donor data were obtained from the bone marrow transplantation service registry. All data were analyzed between the day of infusion of the bone marrow and day 100 after transplantation. If the patient was readmitted within this time interval, all days of hospitalization were considered in calculating the total length of stay, including the days beyond day 100.

Social workers evaluated all patients included in the bone marrow program, and provided adequate accommodation after discharge from the hospital to patients coming from other regions of the country, those living too far from the hospital or those with transportation difficulties. Every patient had a caregiver (spouse or relative) 24 hours per day during the hospital stay, as well as during the outpatient period. Information on personal hygiene and house cleaning, avoidance of house pets and adequate food preparation was given by our nursing staff to all patients and caregivers before the transplantation. These instructions were gathered in a patient's handbook. Informed consent for the transplantation procedures was obtained from each patient and the study was approved by the ethics committee of FMUSP.

The patients were admitted to the hospital for double-lumen catheter placement, high-dose chemotherapy and bone marrow infusion. They were discharged from the hospital thereafter, unless one or more of the following clinical events occurred: fever and/or clinical infection, severe mucositis, grade 3 vomiting or diarrhea, suspected veno-occlusive disease, bleeding or hemodynamic complications.

After discharge, all patients were followed daily at our specialized outpatient clinic, which was open from 7:00 am to 7:00 pm every day, seven days a week, until engraftment (defined as the first of three days of absolute neutrophil count more than 0.5 x10^9^/l). The patients were given medical evaluations, nursing care, medications (intravenous fluids, antibiotics, immunosuppressive agents or antiemetics), laboratory tests and blood transfusion support as needed. After-hours medical or nursing care was provided at the transplantation ward by the staff on call. Telephone assistance was provided at any time. Patients were readmitted to the ward whenever one or more of the following situations occurred: fever unresponsive to broad-spectrum antibiotics, hypotension, severe vomiting or diarrhea, grades III or IV acute graft versus host disease, suspected veno-occlusive disease or severe mucositis in need of parenteral nutrition; or whenever considered necessary by the physician.

Our data were compared with a historical control that consisted of 49 CML patients who underwent transplantation between October 1997 and October 2000 in the same institution. These patients were kept in single air-filtered rooms and were discharged from hospital only after engraftment, thus forming an inpatient group. Forty-seven patients in the inpatient group were in the first chronic phase and two were in the accelerated phase of the disease.

All patients in the inpatient and out-patient groups received bone marrow from HLA-matched (6/6) sibling donors, except for one patient in each group who received partially matched (5/6) HLA sibling donor bone marrow. All patients in both groups were followed at our institution until day 100 post-transplantation.

### Conditioning and prophylaxis

More than 90% of the patients in both groups received busulfan 4 mg/kg/day orally in divided doses daily for four days and melphalan 140 mg/m^2^ intravenously on day −1 as a conditioning regimen (BU-MEL). Two children in the inpatient group and three in the outpatient group received total body irradiation (TBI) and melphalan (TBI-MEL) and one adult in the outpatient group received busulfan and cyclophosphamide (BU-CY)_1_ as conditioning regimens. The graft-versus-host disease prophylaxis included cyclosporin 12 mg/kg/day orally, and methotrexate 15 mg/kg/day intravenously on day 1 and 10 mg/kg/day on days 3, 6 and 11 post-transplantation. The antibiotic prophylaxis included cefepime (whenever the absolute neutrophil count was below 0.5 x 10^9^/l), sulfamethoxazole-trimethoprim (from day −10 to day −1 and thereafter neutrophil recovery), acyclovir and fluconazole. *Cytomegalovirus* antigenemia assaying was performed twice weekly, and preemptive ganciclovir treatment was started whenever two or more positive cells were detected. The inpatients received the same graft-versus-host disease prophylaxis, infection prophylaxis and antiemetics regimen as did the outpatient group. The demographic data are shown in Table 1.

**Table 1.** Inpatient and outpatient group characteristics of chronic myeloid leukemia patients

Characteristics	Inpatients	Outpatients	p
**Patient -** n	49	51	
**Sex -** n (%)			
Male	25 (51.0)	27 (52.9)	1.000
Female	24 (49.0)	24 (47.1)	
**Age in years** - median (range)	32 (10-53)	36 (4-51)	0.173
**Race** - n (%)			
Caucasian	37 (75.5)	31 (60.8)	0.173
Others	12 (24.5)	20 (39.2)	
**Pretransplantation risk factors**^18^ - n (%)			
0-2	39 (79.6)	38 (74.5)	0.174
3-4	10 (20.4)	13 (25.5)	
**Prior therapy -** n (%)			
Hydroxyurea	37 (75.5)	42 (82.4)	0.552
Others	12 (24.5)	9 (17.6)	
**Conditioning** - n (%)			
BU-MEL	47 (95.9)	47 (92.1)	0.787
TBI-MEL	2 (4.1)	3 (5.9)	
BU-CY	-	1 (1.9)	
**Disease stage** - n (%)			
Chronic phase	47 (95.9)	45 (88.3)	0.269
Accelerated phase	2 (4.1)	4 (7.8)	
Blastic phase	-	2 (3.9)	
**Mononuclear cells infused** (x 10^8^) Median			
Median (range)	2.04 (0.9-3.6)	2.10 (0.6-8.0)	0.787

*BU-MEL = busulfan and melphalan; TBI-MEL = total body irradiation plus melphalan; BU-CY = busulfan and cyclophosphamide.*

### Statistics

Comparisons between quantitative variables were made using the Mann-Whitney test, and comparisons between qualitative variables were made using the chi-squared or Fisher tests. Survival rates were compared using the Kaplan-Meier method with the log-rank test. A p-level of 0.05 was considered statistically significant.

## RESULTS

There were no significant differences between the inpatient and outpatient groups in relation to the patients’ sex, race, age, therapy prior to transplantation, risk factors for hematopoietic stem cell transplantation for treating CML,^18^ conditioning regimens or disease stage. The number of mononuclear cells infused, time taken to achieve engraftment and number of days with absolute neutrophil count less than 0.5 x 10^9^/l were not significantly different between the inpatient and outpatient groups. The same was observed regarding veno-occlusive disease, *Cytomegalovirus* (CMV) antigenemia, numbers of packed red blood cell and single-donor platelet concentrate units transfused and acute graft-versus-host disease.

The median start of acute graft-versus-host disease was day 25 (range 13-49) in the inpatient group and day 22 (range 16-44) in the outpatient group. Nausea and vomiting grades 2-3 were significantly more frequent in the inpatient group than in the outpatient group (Table 2). No significant differences were observed in analyzing mucositis and diarrhea. Thirty-eight patients (77.6%) in the in-patient group and 45 (90.2%) in the outpatient group received granulocyte colony-stimulating factor (G-CSF) (p = 0.147). G-CSF was used in neutropenic patients who presented persistent fever or documented infection. No significant differences were observed in relation to catheter and urinary tract infections, pneumonia, sepsis or blood cultures.

**Table 2.** Comparison of outcomes between inpatient and outpatient groups of chronic myeloid leukemia patients that underwent allogeneic bone marrow transplantation

Characteristics	Inpatients	Outpatients	p
**Absolute neutrophil count < 0.5 x 10^9^/l - number of days** - median (range)	14 (7-23)	14 (7-27)	0.837
**Engraftment - time taken to achieve, in days** - median (range)	21 (13-33)	20 (14-35)	0.348
**Acute graft-versus-host disease** - n (%)			
Grade 0-I	16 (32.7)	20 (39.2)	0.635
Grade II-IV	33 (67.3)	31 (60.8)	
**Cytomegalovirus antigenemia assay** - n (%)			
Positive	36 (73.5)	38 (74.5)	1.000
Negative	13 (26.5)	13 (25.5)	
**Transfusions** - Median (range)			
number of red blood cell units	13 (1-85)	12 (0-45)	0.748
number of single-donor platelet units	17 (1-88)	11 (2-66)	0.777
**Fever** - n (%)			
Yes	42 (85.7)	41 (80.4)	0.784
No	7 (14.3)	10 (19.6)	
**Nausea and vomiting** - n (%)			
Grade 0-1	11 (22.4)	32 (62.7)	< 0.001
Grade 2-3	38 (77.6)	19 (37.3)	
**Granulocyte colony-stimulating factor** - n (%)			
Yes	38 (77.6)	46 (90.2)	0.147
No	11 (22.4)	5 (9.8)	

Analysis of bacteriological cultures from different specimens identified *Pseudomonas aeruginosa* infection in 14 inpatient (28.6%) and three outpatients (5.8%) (p = 0.006) (Table 3). Although not significant, more Gram-negative pathogens were identified in the inpatient group and more Gram-positive pathogens in the outpatient group. Most of the Gram-negative infections in the inpatient group were nosocomial infections. Only one *Aspergillus* infection in each group was observed. Fever occurred in 42 patients (85.7%) in the inpatient group and 41 (80.4%) in the outpatient group (p = 0.784).

**Table 3.** Comparison of infections between inpatient and outpatient groups of chronic myeloid leukemia patients

Characteristics	Inpatients	Outpatients	p
**Catheter** - n (%)			1.000
Yes	5 (10.2)	6 (11.8)	
No	44 (89.8)	45 (88.2)	
**Urinary tract** - n (%)			0.205
Yes	9 (18.4)	4 (7.8)	
No	40 (81.6)	47 (92.2)	
**Sepsis** - n (%)			0.227
Yes	3 (6.1)	8 (15.7)	
No	46 (93.9)	43 (84.3)	
**Pneumonia** - n (%)			0.465
Yes	13 (26.5)	18 (35.3)	
No	36 (73.5)	33 (64.7)	
**Blood culture** - n (%)			0.960
Positive	18 (36.7)	20 (39.2)	
Negative	31 (63.3)	31 (60.8)	
***Pseudomonas aeruginosa*** - n (%)			**0.006**
Yes	14 (28.6)	3 (5.9)	
No	35 (71.4)	48 (94.1)	

**Figure 1:**
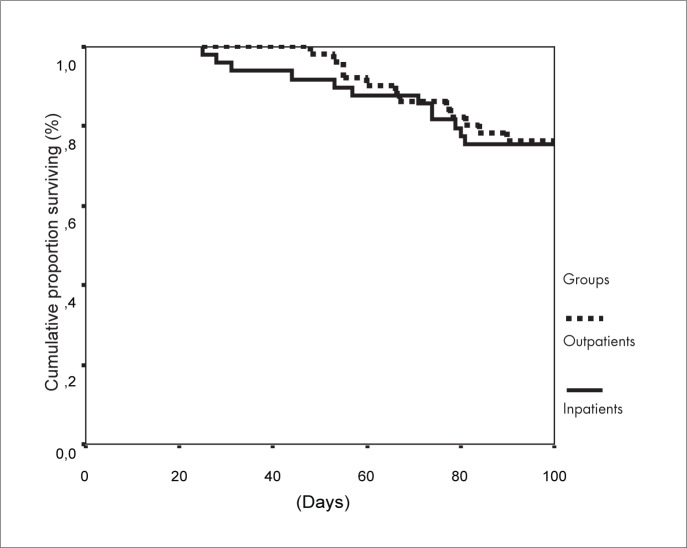
100-day survival after bone marrow transplantation among 51 outpatients and 49 inpatients with chronic myeloid leukemia.

The patients in the inpatient group were discharged after a median of 26 days (range 14-81), and patients in the outpatient group after a median of 6 days (range 1-35 days). In the outpatient group, eight patients could not be discharged early because of clinical complications. The median total length of hospital stay due to the transplantation (including re-admissions) was 28 days (range 16-81 days) for the inpatient group and 17 days (range 1-149 days) for the outpatient group (p = 0.004). Hospital readmission was necessary for 32 outpatients (62.7%), with a median length of stay of 12 days (range 1-75). Twelve deaths occurred in each group (p = 0.910). Deaths in the inpatient group occurred at a median of 64 days after the transplantation (range 25-89), while those in the outpatient group occurred at a median of 66 days after the transplantation (range 48-90) (Figure 1). In both groups, pneumonia and sepsis were the main cause of death, and they were associated with acute graft-versus-host disease grades III/IV.

## DISCUSSION

Our data confirm previous reports that patients who underwent allogeneic hematopoietic stem cell transplantation could be maintained under outpatient-based management during the neutropenic and thrombocytopenic phases without worsening morbidity and mortality.^16,17^ According to other centers that have developed programs for partially or totally outpatient-based hematopoietic stem cell transplantation (autologous or allogeneic) the risks of infection and mortality were not higher among patients who were kept outside of isolated air-filtered rooms or outside of hospital. In our center, the number of deaths was the same in both groups and no early deaths occurred in the outpatient group before engraftment. Nevertheless, this type of transplantation is possible only with a day-hospital that is open seven days a week so that patients can receive all the medical care they need.

In this report we analyzed only patients with the same disease, so that we could eliminate some variables. Previous reports on outpatient allogeneic hematopoietic stem cell transplantation described results relating to different diseases, with various pretransplantation treatments, conditioning regimens and prognostic factors.^9,16,17^ In fact, no significant differences between our inpatient and out-patient groups were detected, such as sex, age, risk factors for transplantation in cases of CML, previous treatment and conditioning regimens. The same could be seen for the median number of mononuclear cells infused, time taken to achieve engraftment and number of days with absolute neutrophil count less than 0.5 x 10^9^/l.

The difference in nausea and vomiting observed in our study, with fewer cases of grade 2-3 in the outpatient group could be explained by the fact that patients could eat food of their preference at home, prepared in the manner they were used to, at the time they wanted. Some previous reports detected more nausea and vomiting among outpatients.^19,20^ Others observed that patients who remained in their homes after allogeneic hematopoietic stem cell transplantation needed less parenteral nutrition, probably because at home they were more active, had better appetites, more motivation to eat and could eat what they were used to, whenever they wanted.^17^

The amounts of packed red cell and single-donor platelet units transfused in the two groups were not significantly different. In some series of outpatient transplantations, more units of red blood cells were transfused to inpatients,^12,17^ while other series used more platelet concentrates in outpatients.^5^

Early discharge did not seem to influence the incidence of acute graft-versus-host disease in our patients, since we could not detect significant differences between the two groups. On the other hand, Svahn et al.^17,21^ detected less acute graft-versus-host disease of grades II-IV in outpatients who underwent allogeneic hematopoietic stem cell transplantation. Although not significant, more cases of sepsis were seen in outpatients. However, most of them occurred later, after engraftment, probably as a consequence of immunosuppressive treatment for acute graft-versus-host disease.

Gram-positive and Gram-negative pathogens were equally isolated from blood cultures in these cases. It was also seen, as previously reported, that blood cultures were negative in most patients with febrile neutropenia.^22,23^ More Gram-negative isolates from blood cultures were observed in the inpatient group and more Gram-positive isolates in the outpatient group, and it is possible that early discharge contributed towards fewer *Pseudomonas aeruginosa-* positive cultures on different specimens from outpatients. Broad-spectrum prophylactic antibiotics were insufficient for decreasing the incidence of episodes of fever and infection in our outpatient group, although less infection was detected in other studies, in outpatients who underwent autologous^24^ or allogeneic^17^ hematopoietic stem cell transplantation. *Aspergillus* infection was not detected more frequently in our outpatient group, although these patients were not kept in air-filtered rooms. As reported by others, there was less occupation of hospital beds by the outpatient group, even when readmissions and the fact that not all patients in our outpatient group could be discharged early were taken into account. Many other centers where allogeneic or autologous transplantations in an outpatient setting have been performed reported less occupation of hospital beds, compared with patients who remained in hospital.^5,9,12,17,24^

## CONCLUSION

Early discharge of chronic myeloid leukemia patients who underwent allogeneic hematopoietic stem cell transplantation, with outpatient care in day-hospital facilities was feasible, without worsening morbidity and mortality. In our hands, this procedure allowed less occupation of hospital beds, with an increase in the number of autologous and allogeneic transplants performed per year, in cases of different diseases, thus making it possible for patients to receive transplantations earlier in the course of their diseases.

A prospective randomized study including other diseases is necessary in our institution in order to confirm our results.
